# Modeling of cell signaling pathways in macrophages by semantic networks

**DOI:** 10.1186/1471-2105-5-156

**Published:** 2004-10-19

**Authors:** Michael Hsing, Joel L Bellenson, Conor Shankey, Artem Cherkasov

**Affiliations:** 1 CIHR/MSFHR Strategic Training Program in Bioinformatics, Genetics Graduate Program, Faculty of Graduate Studies, University of British Columbia, Vancouver, British Columbia, V5Z 3J5, Canada; 2 Upstream Biosciences, Inc., Vancouver, British Columbia, V6H 1H2, Canada; 3 Visual Knowledge, Inc., Vancouver, British Columbia, V6H 1H2, Canada; 4 Division of Infectious Diseases, Department of Medicine, Faculty of Medicine, University of British Columbia, Vancouver, British Columbia, V5Z 3J5, Canada

## Abstract

**Background:**

Substantial amounts of data on cell signaling, metabolic, gene regulatory and other biological pathways have been accumulated in literature and electronic databases. Conventionally, this information is stored in the form of pathway diagrams and can be characterized as highly "compartmental" (*i.e*. individual pathways are not connected into more general networks). Current approaches for representing pathways are limited in their capacity to model molecular interactions in their spatial and temporal context. Moreover, the critical knowledge of cause-effect relationships among signaling events is not reflected by most conventional approaches for manipulating pathways.

**Results:**

We have applied a semantic network (SN) approach to develop and implement a model for cell signaling pathways. The semantic model has mapped biological concepts to a set of semantic agents and relationships, and characterized cell signaling events and their participants in the hierarchical and spatial context. In particular, the available information on the behaviors and interactions of the PI3K enzyme family has been integrated into the SN environment and a cell signaling network in human macrophages has been constructed. A SN-application has been developed to manipulate the locations and the states of molecules and to observe their actions under different biological scenarios. The approach allowed qualitative simulation of cell signaling events involving PI3Ks and identified pathways of molecular interactions that led to known cellular responses as well as other potential responses during bacterial invasions in macrophages.

**Conclusions:**

We concluded from our results that the semantic network is an effective method to model cell signaling pathways. The semantic model allows proper representation and integration of information on biological structures and their interactions at different levels. The reconstruction of the cell signaling network in the macrophage allowed detailed investigation of connections among various essential molecules and reflected the cause-effect relationships among signaling events. The simulation demonstrated the dynamics of the semantic network, where a change of states on a molecule can alter its function and potentially cause a chain-reaction effect in the system.

## Background

Interactions among genes, gene products and small molecules regulate all cellular processes involving cell survival, cell proliferation, and cell differentiation among others. Such interactions are organized into complex lattice structures conventionally divided into cell signaling, metabolic and gene regulatory networks in a cell [1]. In recent years, large amounts of information and knowledge on cell signaling networks have been accumulated in the literature and databases [2,3].

Conventionally, this information is highly compartmental: various individual signaling pathways are mostly stored in separated and non-linked diagrams. Traditional pathway diagrams, where molecules are represented as nodes and their interactions are depicted as lines and arrows have significant limitations as they lack spatial and temporal context [4]. Moreover, the critical knowledge of cause-effect relationships among signaling events is not reflected by most conventional approaches for manipulating pathways. Not surprisingly, the current state of pathway representation does not allow of complex investigation of qualitative or quantitative changes in cell signaling networks in response to external perturbations such as bacterial infections. Thus, an adequate computational environment for modeling cell signaling networks is needed for proper biological data integration as well as for simulation and prediction of cellular behaviors [5].

Recently, many models have been proposed for representing, storing and retrieving interactions among various biological entities. BIND [6] and IntAct [7] focus on protein-protein interactions and their resulting complexes. BioCyc [8] developed models for metabolic events and curated metabolic pathways from many organisms. The model developed by aMAZE [9] combines interactions in cell-signaling, metabolic and gene regulatory pathways. In addition, the System Biology Markup Language (SBML) has been developed for representing biochemical reaction networks and for communicating models used for various simulation programs [10]. Programs such as E-cell [11], Gepasi 3 [12] and Virtual Cell [13] use differential equations to represent molecular interactions, and their simulation results are obtained by solving these questions numerically [14]. It should be noted, however, that many cellular processes are sensitive to the stochastic behavior of a small number of molecules, and therefore, the assumptions in differential-equation methods can often be compromised [15]. Several studies have attempted to address the stochastic property of a cell. Vasudeva and Bhalla [16] proposed a hybrid simulation method that combined both deterministic and stochastic calculations. In addition, a stochastic simulator, StochSim [15] represented molecules as individual software objects that interact according to probabilities. Thus, it is feasible to suggest that useful cell signaling simulators should be capable of representing each molecule individually and reflecting the stochastic behavior of molecular interactions in a cell.

### Semantic networks

Recently an artificial intelligence approach known as semantic networks (SN) have gained the attention of the biological community as a potentially powerful tool for organizing and integrating large amounts of biological information [17]. For instance, the semantic network in the Unified Medical Language System (UMLS) was designed to retrieve and integrate biomedical information from various resources [18]. The UMLS semantic network has also been applied and expanded to include information and knowledge from other domains such as genomics [19]. In addition, other studies have suggested a semantic approach where proteins are viewed as "adaptive and logical agents", whose properties and behaviors are affected by other agents in their spatial organization including intracellular compartments and protein complexes [20,21]. Defining the semantics among agents could characterize both local and global behaviors of a system, and therefore, it is potentially useful to apply such approach to study cell signalling in biological systems [21].

**Figure 1:**
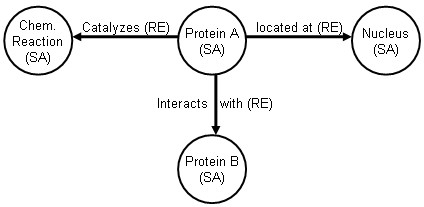
An example of a semantic network. Characteristics and behaviors of a semantic agent (SA) are defined by its relationships (RE) with other agents. Semantic agents are represented as circles, and relationships are depicted as arrows. This SN-model represents that a protein A can be located at a nucleus, can interact with a protein B or catalyze a chemical reaction. For explanatory purpose, this figure illustrates an example of a semantic network. The implemented semantic network (as presented in the paper) is more complex and involves different types of relationships and agents.

A semantic network is a method to represent information or knowledge by nodes and edges in a graphic form, where a node represents a concept and an edge represents a relationship [22]. A semantic network, which can exist abstractly in a human mind or be implemented by applying computer technology, can model many real-world problems [22]. Figure 1 illustrates a semantic network, where a concept such as a protein, a chemical reaction or a subcellular location is modeled by a semantic agent, and its relationships with other agents are represented as arrows. A proper semantic network implementation allows the identity and properties of an agent to arise from its relationships with other agents, not from descriptions or labels [23]. Hence, within a semantic network "things are what they do".

Previously an application development environment known as Visual Knowledge (VK) has been created, and VK is capable of different formalizations and implementations of semantic networks for various knowledge domains [23]. Visual Knowledge has been applied successfully to model and manipulate complex "interactomes", including corporate enterprise systems, flight scheduling networks, hardware maintenance simulators, and integrated currency exchange boards [23]. It has been anticipated that Visual Knowledge can address many of the current limitations on modeling cell signaling pathways. Using the latest VK-based environment, BioCAD [24], specifically designed for biological applications, we have developed a semantic model for cell signaling pathways occurring in human macrophages.

### Bacterial invasions in macrophages

It is the current knowledge that many pathogenic bacteria are capable of entering and surviving within mammalian macrophages by modulating the host signaling pathways [25]. One well-studied example is the activation of the Fcγ macrophage receptor by the IgG antibody, which binds to the surface of bacteria such as *Mycobacterium tuberculosis *[26]. Activation of the Fcγ receptor induces phagocytosis of *M. tuberculosis *and the formation of a phagosome within the macrophage. These processes are mediated by the class I phosphoinositide 3-kinase (PI3K) – one of the most well-characterized enzymes to date [27]. The class I PI3K is a heterodimer composed a p110 catalytic subunit and a p85 regulatory subunit, which maintains a low-level activity of p110 [28]. The p110 subunit is activated when p85 binds at a phosphotyrosine site on a receptor or an adaptor protein, or by direct binding to activated Ras [29]. Activated PI3K-p110 phosphorylates phosphatidylinositol-4,5-bisphosphate (PIP2) into phospatidylinositol-3,4,5-trisphosphate (PIP3), which is an essential signaling molecule that stimulates many downstream proteins, including PDK1 and Akt [30]. The formation of a phagosome is normally followed by the phagosome maturation process, which is responsible for intracellular killing of bacteria and is regulated by the class III PI3K [31]. However, it has been hypothesized that phosphatidylinositol analogs, such as ManLAM, produced by *M. tuberculosis *can inhibit the activity of the class III PI3K, arresting phagosome maturation process, and ensuring the survival of *M. tuberculosis *inside the macrophage [27,32].

In addition to their role in phagocytosis, PI3Ks are essential proteins that regulate cell survival, cell growth, cell cycle and other cellular processes [33]. Although, it is clear that PI3Ks play an important role in bacterial invasions, the knowledge of PI3K-mediated interactions is scattered in a number of literature and pathway databases. A coherent picture of detailed molecular interactions that link receptors to PI3Ks and to various cellular responses has yet to be constructed before bacterial invasions can be fully understood. To address this goal, a cell signaling network of the human macrophage was reconstructed with the semantic model, and qualitative changes in the network were investigated with a SN-simulator.

## Results

### A semantic model for cell signaling pathways

In the paper, the word "model" refers to a set of rules in two different but related contexts. In the context of the semantic network, the model refers to a set of rules that specify how a biological concept is mapped to one or multiple semantic agents/relationships. In the context of cell signaling pathways, the model is a set of rules that specify what, how, and when molecules interact with each other. The model has been formalized and implemented, using BioCAD software system, and it is presented in the following sections.

#### Overall classification of biological structures and their relationships

Within the semantic network, all biological structures are modeled by semantic agents that are members in one of the 6 different prototypes. Table 1 shows the 6 types of structures in the order of their hierarchy. From the highest to the lowest level, they are "Cell", "Subcellular Compartment", "Macromolecule", "Domain/Site", "Small Molecule/Molecular Fragment", and "Atom". A structure agent can be composed of multiple structures of the same prototype or a lower-level prototype, and the agent is connected to its components by the composition relationship in the SN. Thus, a human macrophage has been modeled as a semantic agent of the "Cell" prototype, and it was composed of various "Subcellular Compartment" agents, including plasma membrane, cytosol, nucleus and others. In addition, each compartment such as nucleus contained various agents of the "Macromolecule" prototype including proteins, DNA and RNA. A macromolecule such as a protein was further composed of "Domain/Site" agents like catalytic domains and phosphorylation sites, and a DNA was composed of sites such as promoters and gene regulatory sites.

#### Modeling interactions among biological structures

**Figure 2:**
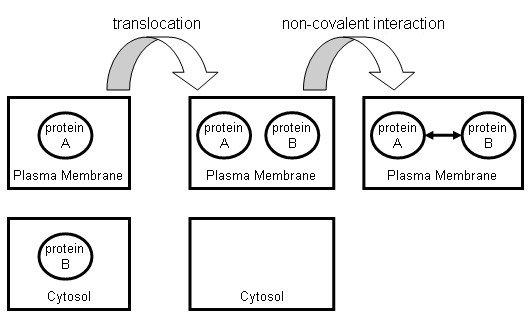
Interactions among biological structures of different levels in the SN. The left panel shows an example of a translocation event when a protein B is moved from the cytosol to the plasma membrane. The right panel shows an example of a non-covalent interaction between a protein A and a protein B via non-covalent forces.

To create an adequate semantic model, we have postulated that structures of different levels in the cellular hierarchy can interact with one another. One example of such interactions is the movement of a molecule from one subcellular compartment (e.g. cytosol) to another (e.g. plasma membrane). This is referred to as a translocation event, and it is demonstrated on the left panel of Figure 2.

Table 2 shows that translocations have been modeled as one the five major "event" prototypes in the SN. Every translocation event has been connected to three structure agents: a molecule to be moved (macromolecule or small molecule), an original location (subcellular compartment), and a destination (subcellular compartment). Hence, the construction of translocation events has enabled us to confine all possible movements of molecules in a cell.

Interactions that occur by non-covalent or covalent forces have also been modeled as two distinct "event" prototypes as shown in Table 2. The right panel of Figure 2 illustrates a general case of a molecular interaction between a protein A and a protein B occurring via non-covalent forces. Such interaction can cause changes of the forms and functions of the interacting molecules, and these changes have been accommodated within the developed SN model by specifying two distinct types of states: "conformational states" and "binding states", also represented by semantic agents.

**Figure 3:**
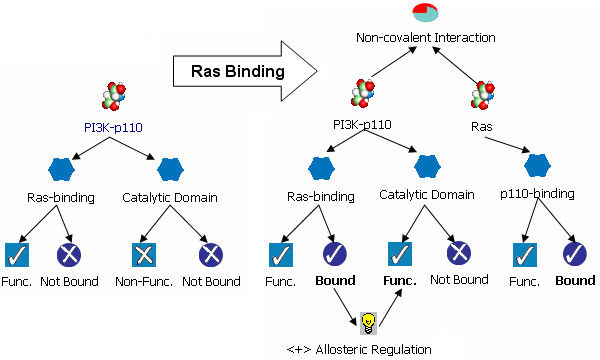
A model of a non-covalent interaction between a PI3K-p110 and a Ras. The figure was graphed from the developed SN to illustrate the relationships among different agents. The figure visualizes the agents as icons and their relationship as arrows. The left panel illustrates that a PI3K-p110 contains a "Not Bound" Ras-binding site and a "Non-Functional" catalytic domain. The right panel shows that when the PI3K-p110 has bound to a Ras, its Ras-binding site has switched to "Bound", and the catalytic domain has become "Functional" due to a positive allosteric regulation event. State changes as a result of the interaction are shown in bold. Note that the model stores the information, which specifies the non-covalent event between the prototypic Ras and the prototypic PI3K-p110, and the condition for the event to occur. This figure illustrates an instance of the Ras-binding event occurred during a simulation. The PI3K-p110 is an instance of the PI3K-p110 prototype, and it is the same agent before and after it binds to the Ras. Figure 8 shows the description of each icon.

All hypothetical spatial changes occurring in the three-dimensional structure of a given macromolecule have been modeled within the SN as switches in the corresponding conformational states, and the changes do not lead to the creation of new semantic agents. Domains or sites for every protein encoded into the SN model have been assigned to either "Functional" or "Non-functional" conformational states. The "Functional" state represents that a domain/site is currently in a conformation that enables a certain interaction. On the other hand, a "Non-functional" state implies a domain/site is in a conformation that prevents an interaction. To illustrate this construct we have graphed the semantic agents and their relationships created within the developed SN. It should be noted that within the SN, all semantic agents are visualized as icons, and their relationships are depicted as connecting arrows. In addition, all agents are related by pairs of reciprocal relationships, and for simplicity, only one direction of each pair of the relationships was visualized. The left panel of Figure 3 features a p110 subunit of the class I PI3K that has been modeled as a "macromolecule" agent and contains a binging site for a Ras protein and a catalytic domain. The Ras binding site has been assigned a state of "Functional", depicted as a check symbol (square) on Figure 3. The "Functional" state enables the PI3K-p110 to bind to a Ras protein. On the other hand, the catalytic domain is "Non-functional', depicted as a cross symbol. Figure 8 shows the description of icons used in this paper.

In addition to the conformational states, a protein domain or site has been assigned one of the two binding states: "Bound" or "Not-bound". A "Bound" state implies that this domain/site currently associates with a domain/site of another molecule through a non-covalent interaction. On the other hand, a "Not Bound" state indicates such an association does not exist. Since ligand bindings can affect the conformation of a macromolecule through allosteric regulations, two types of such regulations have been implemented within the SN. A positive allosteric regulation event has been assigned to the scenario when a "Bound" binding state of a domain/site causes the conformational state of another domain/site to switch to "Functional". The right panel of Figure 3 shows that when the PI3K-p110 has bound to a Ras by a non-covalent interaction, the binding state of the Ras-binding site on p110 has switched to "Bound". As a result, the conformational state of the catalytic domain has switched to "Functional" due to a positive allosteric regulation. The "Functional" catalytic domain now enables the PI3K-p110 to phosphorylate its substrate. On the other hand, a negative allosteric regulation event has been attributed to those cases when a "Bound" state of a domain/site causes the conformational state of another domain/site to switch to "Non-functional". It should be noted that the semantic model stores the information that specifies the non-covalent event between the prototypic Ras and the prototypic PI3K-p110, and the condition for the event to occur. Figure 3 illustrates an instance of the Ras-binding event occurred during a simulation. The PI3K-p110 is an instance of the PI3K-p110 prototype, and it is the same agent before and after it binds to the Ras.

A more complex allosteric regulation event can be specified for mapping the binding states of multiple domains/sites (the condition or the input) to the conformational states of multiple domains/sites (the response or the output). Hence, a domain is switched to "functional" only if a certain combination of ligand bindings has occurred. The utilization of the states on domains/sites and allosteric regulation events in the SN has enabled us to express the cause-effect relationships among various signaling events explicitly.

In the developed semantic model, any molecular complex formed due to non-covalent interaction has been treated as a transient state of these two molecules, and a complex was not represented by a new semantic agent. Instead, the existence of a protein complex is inferred from the non-covalent interaction event. Thus, Figure 3 illustrates a protein complex of the PI3K-p110 and the Ras existed because of the occurrence of the non-covalent interaction event, which connected the two molecules.

**Figure 4:**
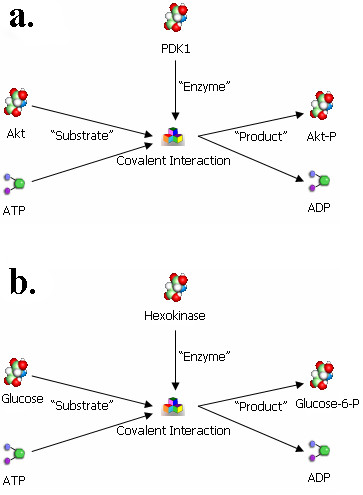
A model for covalent interactions. Figure 4a shows that an Akt protein can be phosphorylated to an Akt-phosphate by an enzyme, PDK1, and an ATP is converted to an ADP in the process. Figure 4b shows a similar covalent interaction event where substrate Glucose can be converted to Glucose-6-phosphate by an enzyme Hexokinase.

Conventionally, there is often some inconsistency between representing chemical modifications of small molecules in metabolic pathways and modifications of proteins in signaling pathways. In the developed model, two molecules that interacted by covalent forces have resulted the creation of distinct semantic agents within the SN. This rule has been implemented consistently for both macromolecules such as proteins and small molecules such as ATP. As one example, Figure 4 features the phosphorylation of an Akt protein by an enzyme PDK1, yielding a distinct Akt-phosphate (Akt-P) agent and a free ADP. Within the SN, the Akt and the ATP are related to a covalent interaction event by "Substrate" relationships, depicted as arrows. In addition, the Akt-P and the ADP are related to the event by "Product" relationships, while the PDK1 is related by the "Enzyme" relationship. The PDK1 enzyme in this example contains a catalytic domain (not shown on the figure), which must be "functional" for the reaction to occur. The state of this domain is under the regulation of the binding of a ligand and an allosteric event as previously defined. In addition, new properties can be assigned to the modified protein. In this case, the phosphorylation by PDK1 switched the catalytic domain in Akt-P to "functional", while this domain was "non-functional" in Akt, the dephosphorylated form. Figure 4 illustrates that a covalent interaction event also applies to metabolites, and a metabolite such as glucose is phosphorylated into a glucose-6-phosphate by an enzyme Hexokinase. Other types of modifications including methylation, acetylation and glycosylation can also be modeled in a similar manner but involve different substrate types.

In the semantic model, a molecule can participate in different sets of interactions in different locations. The translocation events define all possible localizations of molecules, and therefore, an interaction can only occur if the participating molecules can be present in the same location at the same time. Alternatively, an interaction (non-covalent or covalent) can directly associate with a subcellular compartment, and this interaction is only available to molecules in that location.

In addition, all qualitative cellular responses such as cell survival and phagosome formation have been implemented within the SN under a distinct "event" prototype. They have been implemented in a way that the formation or the activation of certain signaling molecules such as PIP3 can trigger the occurrence of these cellular response events in a simulation.

As it has been mentioned previously, the behavior of any semantic agent can be clearly defined by its relationships or connections to other agents. Thus, the formalization of the five types of events, which are translocations, non-covalent interactions, covalent interactions, allosteric regulations and cellular responses, has enabled us to model the behaviors of molecules depicted in conventional pathways and to reconstruct a cell signaling network of the human macrophage.

### Case study: a reconstruction of a cell signaling network in the macrophage

#### Data source

The molecular composition of human macrophages and information of known intracellular interactions have been extracted from various research articles [26,27,32,34-47], review articles [25,28-31,33,48-52] and pathway databases [2,3].

#### Translation and integration of pathway information into the semantic model

A pathway diagram in the literature or an electronic database, in principle, represents some scenario of what may happen in a call if every depicted molecule is expressed in the correct location, at the correct time and with the correct states. Hence, the aggregation of multiple pathway diagrams describes some, if not all, possible molecular events that can potentially occur in a cell under the right conditions. To utilize such information and build a cell signaling network, we have decomposed conventional pathways into individual pieces of information such as subcellular localization of a protein, a pairwise protein binding, a chemical reaction or a cellular response. Then, using the sets of semantic rules described in the model, we have represented and integrated each piece of those information in the form of semantic agents and their relationships. Table 3 illustrates the overall SN model for the cell signaling network contained a total of 93 prototypical macromolecules localized in several subcellular compartments. It included several cell receptors (such as Fcγ, CR3, CD 14, CD18, TLR2) relevant to the process of bacterial internalization of macrophages. Two distinct classes of PI3Ks have been modeled: the class I PI3K composed of p85 regulatory and p110 catalytic subunits, and the class III PI3K composed of p150 and Vps34p subunits [28]. The model also included various kinases such as Lyn, PDK1 and Akt, small GTPases including Ras, Rac1 and Rab5, and adaptor proteins like Gab2. Events of various prototypes have also been extrapolated from the literature and pathway diagrams.

#### Visualization and analysis of the cell signaling network

**Figure 5:**
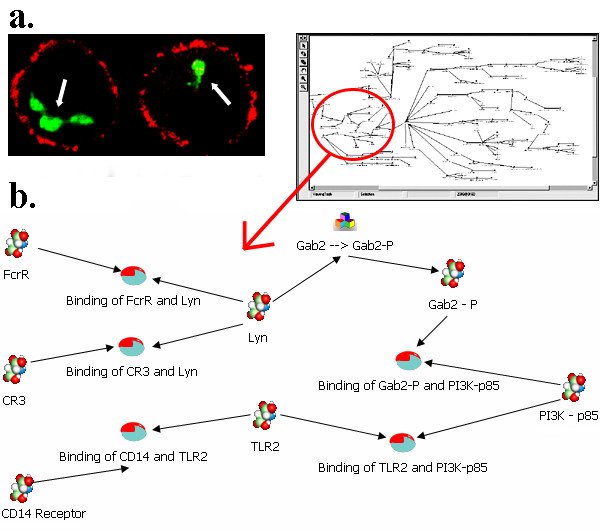
Figure 5a- Phagocytosis of bacteria in macrophages. The picture shows macrophages ingesting green fluorescent mycobacteria (indicated by arrows). The host cell membrane was stained by red fluorochorme PKH to define the limit of the cell. (The picture was provided by Zakaria Hmama) Figure 5b- A SN-representation of the cell signaling network that regulates phagocytosis in the human macrophage. Both molecules and their interactions (non-covalent and covalent interactions) are represented as semantic agents and visualized as nodes (with distinct icons) on the diagram. Arrows represent the semantic relationships between different agents.

The defined semantic agents have been connected in the semantic network and can be visualized at different levels. Figure 5 shows one example of how various non-covalent and covalent interactions have been integrated into a unified cell signaling network. The longest path in the cell signaling network we have created contained 24 consecutive molecular interaction events, linking Fcγ receptor to the class I PI3K enzyme and further through class III PI3K to various cellular responses.

Such detailed semantic reconstruction of the cell signaling network has allowed thorough investigation of biochemical relationships between essential proteins. One such example is presented on Figure 5 featuring the connections among cell receptors Fcγ and CR3, and tyrosine kinase Lyn which they both activate. It has also been reconstructed by the SN model that both of these receptors can activate the class I PI3K via an adaptor protein, Gab2. The corresponding finding will now be subjected to testing in an experimental lab.

Another example of successful SN reconstruction is the relationship between CD14 macrophage receptor and the class I PI3K; such a relationship was previously suspected but not clear [39]. By incorporating the available literature data [35,45] into the semantic environment we were able to reconstruct the scenario where CD14 activates the class I PI3K by the association of Toll-like receptor 2 (TLR2), as it is illustrated in Figure 5. Such model will also be tested experimentally.

### Simulation of changes in the cell signaling network during bacterial invasions

In the implemented semantic model, the "possible" behaviors of a molecule are defined through its relationships to other agents (for example a non-covalent event), and all instances of that prototypical molecule will inherit the same behaviors. However, the action of a molecule at any given time is affected by factors including its current states and its current location with respect to other molecules in the system. Hence, we have built an application that enabled us to produce instances of molecules in various locations and to observe the "current" action of a molecule qualitatively under different biological scenarios. We refer such scenario-play as simulation in this paper.

The application or the SN-simulator allows the molecules to move among various locations, to interact with each other and to create events when the conditions are met. In addition, every instance of a molecule has been represented as an individual agent while every instance of a molecular interaction has also been implemented as an individual event agent. Thus, the simulator provides a traceable "trajectory" of all the events that have happened on every molecule during a simulation.

**Figure 6:**
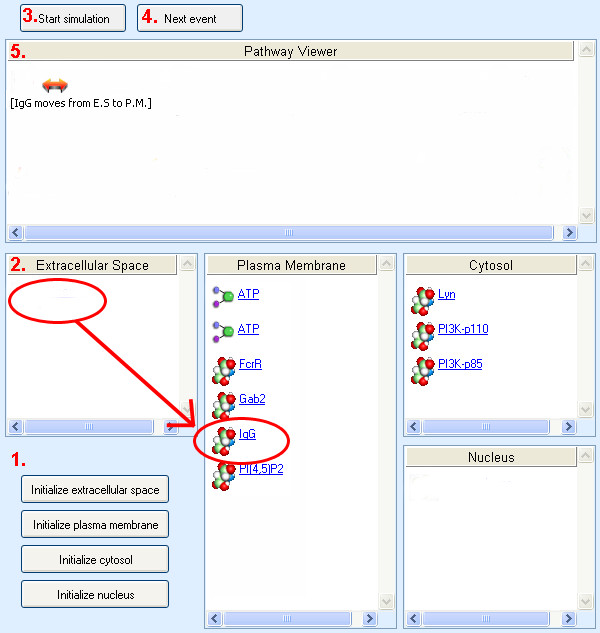
A SN- simulator: at the beginning of the simulation. The simulation showed the actions of molecules under a biological scenario. 1. The initializing buttons synthesize molecules in each subcellular compartment. 2. The localization window shows molecules present in each subcellular compartment. In this simulation, an IgG molecule was present at the extracellular space (E.S.). There were 2 ATP molecules, an Fcγ receptor (FcγR), a Gab2 and a PIP2 (PI[4,5]P2) present at the plasma membrane (P.M.). The cytosol contained a Lyn kinase, a PI3K-p85 and a PI3K-p110 subunit. There was no molecule present at the nucleus in this simulation. 3. The "Start Simulation" button creates a previously specified translocation event. In this simulation, the translocation has already occurred and moved the IgG from the extracellular space to the plasma membrane. 4. The "Next" button triggers a search that determines a proper event to occur and advances to the next step. 5. The pathway-viewer shows a series of events occurred during the simulation.

As illustrated in Figure 6, the macrophage cell has been generally divided into four subcellular compartments or locations within the simulator. We have specified what molecules to be present initially in each subcellular location in the beginning of a simulation, and the simulator synthesized molecules in each location accordingly. At the very first simulation step, the simulator has created a translocation event moving a molecule (the current target) from one location to another. The initial translocation has been specified as the movement of an IgG molecule from the extracellular space to the plasma membrane as shown in the pathway-viewer on Figure 6. The occurrence of this initial event allowed the simulator to trigger a search and advanced to the next step. The search looked for other potential molecules (with the correct states) that can interact with the target molecule in the same location. If multiple instances of potentially interacting molecules were present in that location, a single molecule would be randomly selected to interact with the target.

**Figure 7:**
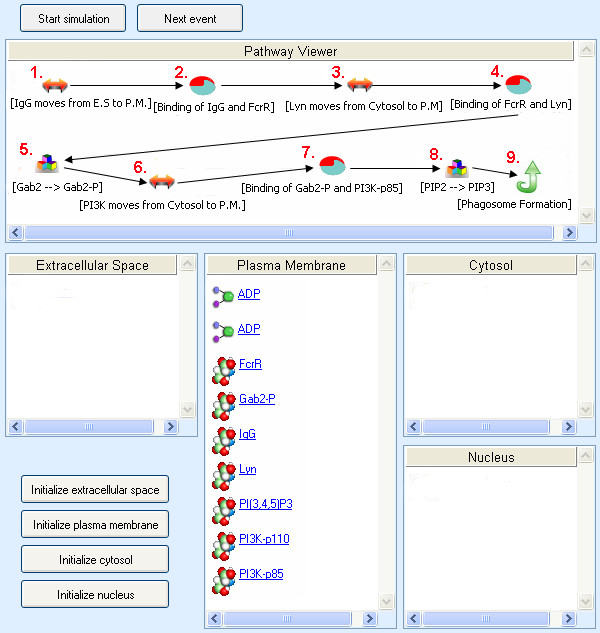
A SN- simulator: at the end of the simulation. The pathway-viewer shows that the initial translocation of the IgG molecule has led to the occurrence of a series of events, which include several non-covalent interactions, covalent interactions, and translocations of various molecules: Event #1: the IgG was translocated from the extracellular space to the plasma membrane. Event #2: the IgG bound to the Fcγ receptor at the plasma membrane. Event #3: the Lyn was translocated from the cytosol to the plasma membrane. Event #4: the Lyn bound to the Fcγ receptor at the plasma membrane. Event #5: the Lyn phosphorylated the Gab2 to a Gab2-phosphate (Gab2-P) at the plasma membrane. Event #6: the PI3K-p85 and p110 (already bound to each other) were translocated together from the cytosol to the plasma membrane. Event #7: the PI3K-p85 bound to the Gab2-P at the plasma membrane. Event #8: the PI3K-p110 phosphorylated the PIP2 to a PIP3 (PI[3,4,5]P3) at the plasma membrane. Event #9: The formation of the PIP3 caused phagosome formation.

**Figure 8:**
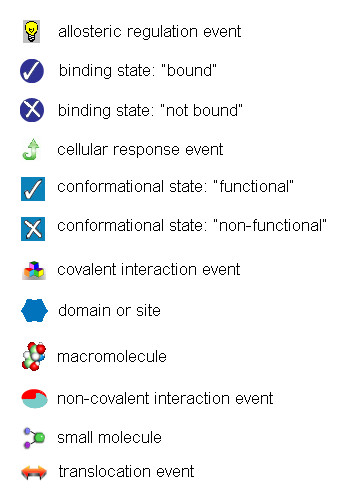
Description of icons used in other figures.

Because an Fcγ receptor was the only interacting molecule (for the IgG) present at plasma membrane in the simulation, it has bound to the IgG by a non-covalent interaction event, as illustrated in Figure 7. This non-covalent interaction has switched the state of the Fcγ receptor's binding site for a Lyn kinase to "Functional", and thus it enabled the Fcγ receptor to bind to a Lyn. However, the Lyn was not initially present in plasma membrane, but it was localized in cytosol in the beginning of the simulation, as shown in Figure 6. Thus, when the Lyn has been translocated from the cytosol to the plasma membrane, a non-covalent interaction between the Lyn and the "Functional" Fcγ receptor occurred in the following step as shown in Figure 7. The search was iterated and the simulation continued until all interacting molecules have been depleted in the macrophage.

Figure 7 demonstrates the consecutive events in this simulation scenario where the Lyn protein phosphorylated a Gab2, which then bound to a class I PI3K. When activated, the PI3K phosphorylated a PIP2 into a PIP3, which in turn caused a phagosome formation response. Different setups of the initial localization of molecules have affected the outcome of the simulation. For instance, an initial presence of a Rab5 (a downstream protein of the PIP3) and a class III PI3K in the cytosol extended the previous pathway from the PIP3. This localization setup stimulated a PIP3-mediated activation of the class III PI3K, which led to phagosome maturation response in the simulation. However, if a phosphatidylinositol analog, ManLAM, of *M. tuberculosis *was initially present in the plasma membrane, it would inhibit the class III PI3K and thus arrest the phagosome maturation response in the macrophage. Table 4 shows that the activation of PI3Ks-mediated pathways by *M. tuberculosis *has caused several known cellular responses as well as additional responses such as cell survival of the macrophage, cell cycle entry, increase of protein synthesis and increase of intracellular glucose level in the simulation. We suspect that some of these responses have not yet be appreciated in previous studies of bacterial invasions. Further experiments can be formalized to test the simulation results.

In this study, the SN-simulator has enabled us to "play" different scenarios and observed their effects in the macrophage. It allowed us to investigate how changes on one molecule caused changes of another molecule in the cell signaling network during bacterial invasions.

## Discussion

### Features of the semantic model

In the present work we have developed a semantic model to represent the properties and the behaviors of molecules and their interactions in the context of cell signaling pathways. The proposed model offers some additional features, compared to other existing pathway models. Those features are essential for characterizing the complex behaviors of biological entities, and they include:

#### Specify the spatial organization of molecules

The semantic model has specified the hierarchical relationships among the different biological structures, from cells to compartments, molecules and domains/sites. The hierarchy between subcellular compartments and molecules has allowed us to specify the spatial organization of molecules, model the translocation events and represent the effects of locations on the different interactions among molecules.

#### Model proteins as integrating and logical devices

The hierarchy between molecules and their domains/sites has enabled us to explicitly model the relationship between forms and functions for proteins. Through the allosteric regulation events, proteins have been modeled and implemented as integrating and logical devices in the semantic network, and their conformational states (outputs) are switched by the combination of non-covalent ligand bindings or covalent modifications (inputs).

#### Provide a direct communication from models to simulations

Through the prototyping system in the semantic network, any rule or interaction specified on a prototypical molecule automatically define the properties and behaviors of all its instances. As demonstrated by the simulator, the semantic network provided a direct communication from the interaction model to an application where the actions of molecules can be observed under different scenarios. Therefore, the semantic network is dynamic as a change of states on a molecule can alter its function and potentially cause a chain-reaction effect in the system.

#### Reduce the need for labels

In addition, the current semantic model is different from the previous models in BioCAD. An essential difference is the representation of functional labels or roles on proteins. The meanings of functional descriptions or association words such as "enzyme", "activator/activates" or "inhibitor/inhibits", which are often used to characterize the behaviors of proteins in most pathway models, have been represented explicitly through events and relationships in the developed semantic network. For example, a protein acts as an "enzyme" if 1) the protein participates in a "covalent interaction event", 2) the presence of a "functional" catalytic domain on the protein is required for the occurrence of the event, and 3) the protein itself is not modified after the event. Similarly, a protein A "activates" a protein B if a non-covalent binding event from protein A turns on the "functional" state of a domain/site on protein B. Hence, the model has reduced the need for labels, which are often confusing or misleading on conventional pathway representation.

#### Future directions

The use of non-covalent and covalent events has enabled us to model protein-protein interactions and chemical modifications on molecules including proteins and metabolites. The next challenge is to model the complex interactions that govern gene regulations. The current construction of non-covalent interaction events can model the binding of an individual transcription factor to a particular site of a gene, and the covalent interaction event can represent the transcription process that leads to the production of an mRNA, and the translation process that produces a protein. However, a successful transcription in a eukaryotic cell requires the formation of a protein complex that involves more than one hundred subunits, and the complex may be assembled in various orders [53]. We anticipate the improvement of the current allosteric regulation model to characterize the more complex logic in gene regulation.

The semantic network representation can be exploited for performing analysis of cell signaling pathways. The examples of Fcγ receptor, CR and the class I PI3K demonstrated that connections can be queried and analyzed among different biological entities. The semantic model is also compatible with other pathway models. Therefore, the number of biological entities and interactions in the semantic network can be greatly increased as pathway data from existing databases is integrated. Previous study has shown the value of combining gene expression profiles with protein-protein interaction networks for identifying active subnetworks [54]. Similarly, data from gene and protein expression experiments could be integrated with the semantic network for "pathway filtering". For instance, within a particular cell, there could be multiple paths that connect two proteins, while each path consists of different number of nodes. When the cell receives a signal, the shortest path, the one with the least number of nodes that require activation, is more likely to be "walked" than a longer path. Hence, the gene/protein expression data will provide some estimation of an overall protein expression and activation states to identify "active" pathways in a cell under a given condition

In this study, the proposed semantic model has been applied to cell signaling pathways in the macrophage as a case study. The model is not limited to those pathways. The hierarchical classification of the biological structures and the events can model other cell signaling pathways for different cells and organisms. An interactive website is currently under development. We anticipate that through the web, researchers can utilize the semantic network approach for creating pathways in cells of their interest and for analyzing any existing pathways including the PI3K pathways of the human macrophage presented in the paper.

### The current capability and applicability of the SN simulator

In this study, we have developed a simple simulator to demonstrate the dynamics of the semantic network and to observe the actions of molecules qualitatively. In order to perform a realistic cellular simulation in the future, three components need to be improved. First, quantitative factors should be integrated into the model. For example binding affinity, directly associated with non-covalent events, will affect the probability and the duration of the binding of molecules. Reaction kinetics, associated with covalent events, will determine the rate of production.

Second, the two parameters, the population of molecules and their localization, which influence the simulation outcome, could be initialized and supported by experimental data. For instance, gene expression data from microarrays supports the relative abundance of transcripts, and protein expression data supports the relative abundance of proteins. Computer algorithms such as PSORT [55] can assist in predicting the localization of proteins.

Third, the proximity of molecules has been represented by subcellular compartments in the simulation. This approximation can be improved in two different ways. First, a compartment can be further divided into smaller sub-locations. Increasing the number of locations and reducing the size of each location will improve the accuracy of the simulation. Second, the occurrence of non-covalent events in the simulation has allowed us to identify molecular complexes and their members effectively. Hence, the proximity can be approximated through molecular complexes, such that molecules in a complex have higher probability to interact with members of the same complex.

The simulator has demonstrated that a biological pathway can emerge from the creation of semantic agents and their relationships in the SN, and such a pathway represents a series of consecutive events resulting from the activation of a single molecule. It is anticipated that further development will improve our ability to track and visualize different instances of molecules participated in multiple pathways. Hence, the occurrence of a cellular response event can be triggered by the accumulation of certain molecular species with particular states.

## Conclusions

We concluded from our results that the semantic network is an effective method to model cell signaling pathways. Utilizing the semantic agents and the relationships in the model, information on biological structures and their interactions at different levels has been properly represented and integrated in the hierarchical and spatial context. The reconstruction of the cell signaling network in the macrophage has allowed qualitative investigation of connections among various essential molecules and reflected the cause-effect relationships among the events. The simulation demonstrated the dynamics of the semantic network, where actions of molecules are affected by their current states and locations, and the history of events can be traced and analyzed. In addition, changes caused by the invading *M. tuberculosis *in the macrophage were investigated by the simulator. As a result, the simulation identified pathways of molecular interactions that led to known cellular responses as well as other potential responses during bacterial invasions.

## Methods

### The Visual Knowledge environment

Visual Knowledge (VK) is an application development environment, and its implementation has been influenced by the theory of semantic networks as well as other approaches including set theory, frame system, object-oriented modeling theory and systems based on networks of active software agents [23]. Different from other passive knowledge representation technology, VK is dynamic and scalable, and it is capable of active representation and integration of different domain knowledge. By manipulating a number of fundamental classes of semantic agents like "physical thing", "event" and "trigger", models of various complexity can be constructed with VK. In addition, VK allows the creation of "prototypes" within each basic class of agents, and therefore it enables any classification of agents based on their common characteristics and behaviors.

### The BioCAD software

BioCAD, a Visual Knowledge-based development environment, is developed by Upstream Biosciences, Inc. and customized to model biological systems [24]. The BioCAD software provides tools for managing large-scale biological data and for visualizing and editing biological pathways and networks. BioCAD currently contains millions of biological concepts and hundreds of pathways that have been integrated and curated from publicly available data sources. A locally installed client program allows semantic agents to be created, stored and queried from a remote central server. The BioCAD software is available commercially, and a collaborative modeling server will be publicly accessible soon.

## Authors' contributions

The semantic model was developed jointly by all authors and implemented by MH, JLB and CS. MH implemented the simulation, collected and analyzed data, constructed pathways in the macrophage and drafted the manuscript. JLB, CS, AC developed general concepts, provided scientific support, participated in the manuscript writing and coordinated the study. All authors read and approved the final version of the manuscript.

## Figures and Tables

**Table 1:** Classification of biological structures in 6 prototypes in the semantic network.

**Semantic Agent – Structure**	**Biological Example**
Cell	Human macrophage, *Mycobacterium tuberculosis*
Subcellular Compartment	Plasma membrane, cytosol, phagosome, nucleus
Macromolecule	Protein, nucleic acid, polysaccharide, fat/lipid
Domain and Site	Catalytic domain, SH2 domain, PH domain, binding site, phosphorylation site, promoter, gene regulatory site.
Small Molecule and Molecular Fragment	Amino acid, nucleotide, sugar, fatty acid
Atom	Hydrogen, carbon, oxygen, nitrogen, phosphorus, sulfur

**Table 2:** Classification of biological events in 5 prototypes in the semantic network.

**Semantic Agent – Event**	**Biological Example**
Translocation	A protein moves from cytosol to plasma membrane.
Non-covalent Interaction	A ligand binds to a receptor.
Covalent Interaction	An enzyme catalyzes a chemical reaction where substrates are converted to products.
Allosteric Regulation	A ligand binding on site A of a protein causes a conformational change on site B of the protein.
Cellular Response	Cell survival, cell death, phagosome formation, increase of intracellular glucose level.

**Table 3:** The number of structure and event prototypes modeled in the cell signaling network of the macrophage.

**Structure**	**Sum**
Subcellular Compartment	9
Macromolecule	93
Domains and Site	20
Small Molecule	9
**Event**	**Sum**

Translocation	24
Non-covalent Interaction	57
Covalent Interaction	28
Cellular response	22

**Table 4:** Simulation results from *M. tuberculosis *invasion in the human macrophage.

**Known response in the macrophage**	**Other potential response in the macrophage**
Actin Polymerization	Cell survival
Pseudopod Extension	Cell cycle entry – S phase
Phagosome Formation	Increase of protein synthesis
Phagosome Maturation Arrest	Increase of intracellular glucose level
